# Prevalence and determinants of adverse perinatal outcomes of preeclampsia with severe features at two selected public hospitals in Addis Ababa, Ethiopia

**DOI:** 10.3389/fped.2024.1345055

**Published:** 2024-02-08

**Authors:** Mesfin Tadese, Wogene Asefa Damesa, Gebeyehu Shumet Solomon, Girma Wogie Fitie, Yohannes Moges Mitiku, Saba Desta Tessema, Agizew Endale

**Affiliations:** ^1^ Department of Midwifery, School of Nursing and Midwifery, Asrat Woldeyes Health Science Campus, Debre Berhan University, Debre Berhan, Ethiopia; ^2^ Department of Medicine, Obstetrician and Gynecologist, Abebech Gobena Mothers and Childrens Health Hospital, Addis Ababa, Ethiopia; ^3^ Department of Epidemiology, St. Peter Specialized Hospital, Addis Ababa, Ethiopia; ^4^ Department of Nursing, Debre Berhan Health Science College, Debre Berhan, Ethiopia

**Keywords:** preeclampsia, preeclampsia with severe features, perinatal outcomes, factors, Ethiopia

## Abstract

**Background:**

Preeclampsia is a new onset of hypertension and either proteinuria or end-organ failure after 20 weeks of gestation. It is a prevalent cause of perinatal mortality, morbidity, and neonatal complications in developing nations including Ethiopia. Thus, the aimed to assess the prevalence and determinants of adverse perinatal outcomes among women with preeclampsia with severity features (PEWSF) at two selected public hospitals in Addis Ababa, Ethiopia, 2023.

**Method:**

A cross-sectional study was carried out among 348 mothers between January 1, 2023, and July 1, 2023. A structured, pre-tested questionnaire was used to gather data from in-person interviews and a review of the patient's medical record. The statistical program Epi-Data version 4.6 was used to enter the data, and SPSS version 26.0 was used for analysis. Binary logistic regression analysis was used to find factors that were associated with unfavorable perinatal outcomes. A *p*-value of less than 0.05 was used to declare the significance level.

**Result:**

The overall prevalence of unfavorable perinatal outcomes was 59.2% (95% CI: 54.0–63.8). Among the complications, low birth weight, prematurity, NICU admission, and a low fifth-minute APGAR score, encompass 48.9%, 39.4%, 20.4%, and 14.7%, respectively. No formal education [OR = 5.14, 95% CI: (1.93–13.63)], unemployment [OR = 0.42, 95% CI: (0.24–0.73)], referral cases [OR = 2.03, 95% CI: (1.08–4.06), inadequate antenatal care (ANC) contact [OR = 3.63, 95% CI: (1.22–10.71)], and family history of hypertension [OR = 1.99, 95% CI: (1.03–3.85)] have shown a statistically significant association with unfavorable perinatal outcomes.

**Conclusion:**

In this study, the prevalence of unfavorable perinatal outcomes was high compared to other studies in Ethiopia. Level of education, occupation, mode of admission, ANC contact, and family history of hypertension were significant predictors of unfavorable perinatal outcomes. Socio-economic development, improving referral systems, and adequate antenatal care contact are needed to improve unfavorable outcomes. Additionally, antenatal screening and specialized care for high-risk mothers, e.g., those with a family history of hypertension are recommended.

## Background

Preeclampsia is a new occurrence of hypertension accompanied by either end-organ failure or proteinuria following 20 weeks of gestation, during pregnancy, delivery, or the postpartum period (1). It is most likely caused by a combination of maternal and fetal/placental factors. Early in pregnancy, abnormalities in the placental vasculature may cause relative placental hypoxia, ischemia, or under-perfusion (2). This, in turn, may trigger the release of antiangiogenic factors into the mother's circulation, which modifies the function of the mother's systemic endothelium and results in hypertension as well as other disease manifestations (hematologic, neurologic, cardiac, pulmonary, renal, and hepatic dysfunction) (3). The cause of aberrant placental development and the series of events that follow, however, is yet unknown (4).

In high-income nations, preeclampsia complicates between 3% and 5% of pregnancies (5). In Africa, five to ten percent of pregnancies are affected with hypertensive disorders during pregnancy (6). According to a study conducted in Zanzibar, 26.3% of mothers had preeclampsia with severe features (PEWSF) (7). A systematic review and meta-analysis study found that the pooled prevalence of preeclampsia in Ethiopia is 11.5% (8). Furthermore, a prospective observational study conducted at Saint Paul's Hospital Millennium Medical College in Ethiopia found 19.5% of cases of preeclampsia with severe features (9).

In the USA, 12% of pregnancies complicated by preeclampsia with severe features resulted in adverse newborn outcomes (10). Besides, in Nigeria, 63.5% of preeclamptic mothers have unfavorable perinatal outcomes (11). Women in Southern Ethiopia with severe features of preeclampsia had a 46% higher risk of unfavorable perinatal outcomes (12). The proportion of adverse perinatal outcomes of severe preeclampsia in Ethiopian referral hospitals was found to be 46.6% (13). Additionally, about 66.4% of mothers with PEWSF in Addis Ababa, Ethiopia, experienced at least one neonatal complication (14).

Because of the progressive nature of the disease and the lack of a recognized medical treatment, delivery is the proven treatment, yet when to deliver is a topic of discussion for both preterm and term gestations (15). Prolonging pregnancy increases the chance of aggravating the mother's endothelial dysfunction and maintaining insufficient blood flow to the target organs, which can cause major harm to the brain, liver, kidneys, placenta/fetus, hematologic, and vascular systems (1). As a result, pregnant women with preeclampsia had a greater chance of premature birth (67.7%) (16). In the USA, preeclampsia also decreased the gestational age at delivery by nearly two weeks (17). Stillbirth, respiratory distress syndrome, intrauterine growth restriction, low birth weight, birth asphyxia, and admission to the intensive care unit are also possible consequences for neonates (14, 18). Additionally, a lifelong risk of hypertension and chronic renal disease was reported for both the mother and child (19). Children and adults born to preeclamptic women are more likely to experience mental health disorders and cognitive impairments (20). Further, hypertensive disorders of pregnancy significantly contributed to postpartum maternal deaths (21).

A study in Ethiopian Referral Hospitals identified the risk factors strongly associated with unfavorable perinatal outcomes as educational status, parity, gestational age, and time of drug given to the mother (13). The estimated gestational age at admission, mode of delivery, and severity of the disease had also a positive association with unfavorable perinatal outcomes (11). Further, educational status, maternal parity, history of ANC follow-up, having preeclampsia and eclampsia, and late provision of drugs, e.g., antihypertensive, anticonvulsant, and corticosteroid were the independent factors significantly associated with adverse perinatal outcomes (22).

Following the World Health Organization, the Ethiopian government has made efforts to curb the high burden of perinatal death by putting in place various measures at both facility and community levels. The key interventions were preventing or treating the primary causes of neonatal and child mortality, such as providing essential immunizations, treating and preventing malaria, and vitamin A supplementation, encouraging institutional delivery, offering 24-h maternity services, including ambulance access, spacing births, increasing the use of family planning, educating health extension workers, encouraging early and exclusive breastfeeding, and enhancing socioeconomic conditions (23). Moreover, Ethiopia recently switched to the new ANC eight-contact model from the outdated four-visit targeted prenatal care (ANC) approach (24). In Ethiopia, there was a notable reduction in under-5 and infant mortality (decreased from 123 to 77 per 1,000 live births in 2005 to 59 and 47, respectively, in 2019. However, there have been no significant reductions in perinatal mortality since 2016 (33 deaths per 1,000 live births in 2016 and 2019) (25). Globally, 500,000 babies dies from the preeclampsia each year (26). A prospective cohort study conducted in Southern Ethiopia also found that preeclampsia contributed to higher rates of early neonatal deaths (12). Worldwide perinatal mortality from severe preeclampsia/eclampsia continues to rise despite intense efforts to minimize pregnancy-related complications and mortalities (11, 14).

Few studies have been conducted to date on the adverse perinatal outcomes among pregnant women with preeclampsia with severe features in underdeveloped nations, such as Ethiopia. The finding could have its own slight share in achieving the target set at the local [Ethiopian Health Sector Transformation Plan-II (HSTP-II)] targeted to lower newborn mortality from 33 to 21 per 1,000 live births by 2025 (27) and global level [Sustainable Development Goal (SDG)] target plans to reduce the global neonatal mortality rate to at least as low as 12 per 1,000 live births by 2030 (28, 29). Thus, the study aimed to determine the prevalence and determinants of adverse perinatal outcomes among mothers of preeclampsia with severe features in Ethiopia.

## Methods

### Study design, setting, and period

A prospective cross-sectional study was carried out in Addis Ababa, the capital city of Ethiopia, from January 1, 2023, to July 30, 2023, at Abebech Gobena Mothers and Childrens Health (MCH) and St. Peter's Specialized Hospital. One of the tertiary referral hospitals directly under the Addis Ababa Health Bureau is Abebech Gobena MCH Hospital. It serves as a teaching hospital for Yekatit 12 Hospital Medical College as well. Every year, the hospital treats about 200,000 patients who were referred by roughly 18 primary hospitals, as well as catchment health clinics throughout the Oromia regional state and Addis Ababa city. On the other hand, the first tuberculosis (TB) referral hospital in the country was a government establishment called St. Peter's Specialized Hospital. In 1953, the hospital was established. presently serving as a specialist hospital overseen by the Federal Ministry of Health (FMOH), offering care to more than 100,000 patients. Serving 15 catchment health centers and 3 primary hospitals from the Oromia region and Addis Ababa city, the MCH center was founded in 2006 E.C.

### Population and eligibility criteria

The source population consisted of all pregnant women diagnosed with preeclampsia severe features in the study area. The study population consisted of mothers who were selected at random and diagnosed with preeclampsia with severe features during the study period. Included were all pregnant women who underwent diagnosis, admission, and management for PEWSF. Mothers who died due to complications from PEWSF and were not giving birth at the study facilities with unclear perinatal outcomes were not included.

### Sample size and sampling technique

Using the assumptions of 36% prevalence of unfavorable outcomes in Addis Ababa, Ethiopia (14), 95% confidence interval, 5% marginal error, and 5% non-response rate, the sample size was calculated using OpenEpi Version 3.03 statistical software. Taking into account the final sample size was 372. The total population sampling method was used to choose study participants.

### Variables

The dependent variable was the perinatal outcome. The study included several independent variables, such as sociodemographic factors; age, marital status, residence, occupation, educational attainment, and mode of admission. Additionally, medical and reproductive history were examined, including gravidity, parity, history of abortion, and antenatal care (ANC), history of pregnancy-induced hypertension, family history of hypertension, anemia, chronic hypertension, diabetes, and renal disease. Other factors included clinical features and investigations upon admission, such as headache, dizziness, epigastric pain, visual disturbance, nausea and/or vomiting, convulsion, edema, liver function test, urea, creatinine, and urine protein. Finally, obstetric factors were considered, including onset of labor, mode of delivery, neonate's sex, and length of hospitalization.

### Definition of terms

#### Preeclampsia with severe features

Refers to preeclampsia with one of the severity features, such as altered mental status, severe headache, altered cerebral or visual disturbance, hepatic abnormality, renal abnormality, severe blood pressure (≥160/110), thrombocytopenia (platelet count <100,000/μl), and pulmonary edema (1, 5, 14).

#### Severe headache

A debilitating headache that is “the worst headache I have ever had” or that worsens and doesn't go away even with analgesic treatment (1).

#### Hepatic abnormality

Severe, ongoing right upper quadrant or epigastric discomfort that is not relieved by medicine and cannot be explained by any other diagnosis or serum transaminase concentration ≥2 times the upper limit of the normal range, or both (1, 14).

#### Renal abnormality

Serum creatinine concentration doubling in the absence of other renal disease, or progressive renal insufficiency as measured by serum creatinine >1.1 mg/dl [97.2 micromol/L] (1, 14).

#### Unfavorable perinatal outcome

Newborns who develop at least one perinatal complication of preeclampsia with severe features, i.e., low 5th min APGAR score, need for resuscitation, neonatal intensive care unit admission, stillbirth/ intrauterine fetal death (IUFD), intrauterine growth restriction (IUGR), prematurity, low birth weight (13, 14).

#### Favorable perinatal outcome

Newborns who did not develop a perinatal complication of preeclampsia with severe features (13, 14).

### Data collection tool, procedure, quality control

A pre-tested, structured questionnaire was used to gather data from in-person interviews and a review of the patient's medical record. The survey was modified based on comparable research (11, 13, 14, 16, 18). There were two supervisors and four data collectors on the data collection crew. The supervisors and data collectors received a one-day instruction from the lead investigators regarding the goals, protocols, techniques, and data-gathering tool. To ensure that the questions stuck to their intended purpose, they were translated back and forth between Amharic and English. At Debre Berhan Comprehensive Specialized Hospital, a pre-test was conducted on 5% of the samples, or 19 women, before the actual data collection. Based on the test results, necessary adjustments were made. The lead investigators and supervisors kept a close eye on the data's completeness, consistency, and clarity throughout the collection process.

### Data management and analysis

The statistical program Epi-Data version 4.6 was used to enter the data, and SPSS version 26.0 was used for analysis. For quality control, the lead investigator chose a questionnaire at random and cross-checked it with the clinical chart and data that matched. To describe the independent and dependent variables, we used descriptive statistics. The number, frequency, percentage, and comparison of perinatal outcomes were displayed as the results. Tables showed the results of the crosstabs using chi-square testing. An independent predictive analysis of unfavorable perinatal outcomes was conducted using binary logistic regression analysis. The final multivariable logistic regression analysis model contained variables from the bivariable regression analysis that had a *p*-value of less than 0.25. The goodness-of-fit test developed by Hosmer and Lemeshow was used to assess the model's fitness and the value was 0.75. Additionally, the value for Nagelkerke R square during the final model was 0.36. Adjusted odds ratios (AORs) were used to interpret the strength of the link, with a two-sided 95% confidence interval (CI). A *p*-value of less than 0.05 was used to declare the significance level.

### Ethical approval

Ethical approval was provided by Yekatit 12 Hospital Medical College's Institutional Review Board (Protocol number 128/23). A formal letter of support was sent to the hospitals under scrutiny. The participants voluntarily participated and gave their free and written informed permission. After the information was read, those who were illiterate were requested to thumbprint the consent form. No payment or special privilege was granted to the study subjects for taking part in the study. However, health education regarding the course, complications, and management options of the disease and preconceptional care for any subsequent pregnancies were provided. After data collection was finished, client records were returned to their original location while maintaining confidentiality and anonymity.

## Result

### Socio-demographic characteristics of participants

There were 348 responders in all, yielding a 93.5% response rate. The mean (±SD) age of participants with favorable and unfavorable perinatal outcomes was 28.15 ± 5.20 and 27.13 ± 5.14, respectively. Of the participants with unfavorable and favorable outcomes, approximately one-third, 70 (34.0%) and 64 (45.1%) had attended secondary education, respectively (*p* = 0.003). Additionally, 187(90.8%) of those who had unfavorable outcomes and 137 (96.5%) of those who had favorable outcomes were married (Table 1).

**Table 1:** Socio-demographic characteristics of mothers admitted with PEWSF at two selected public hospitals in Addis Ababa, Ethiopia, 2023.

Variables	Category	Perinatal outcomes		
		Favorable (142)	Unfavorable (206)	*p*-value
Age	20–34 years <20 years ≥35 years	119 (83.8%) 4 (2.8%) 19 (13.4%)	175 (85.0%) 9 (4.4%) 22 (10.6%)	0.584
Residence	Urban Rural	117 (82.4%) 25 (17.6%)	155 (75.2%) 51 (24.8%)	0.113
Level of education	No formal education Primary Secondary Higher education	10 (7.0%) 32 (22.5%) 64 (45.1 36 (25.4%)	35 (17.0%) 65 (31.5%) 70 (34.0%) 36 (17.5%)	0.003
Marital status	Married Others^a^	137 (96.5%) 5 (3.5%)	187 (90.8%) 19 (9.2%)	0.039
Occupation	Employed Unemployed	77 (54.2%) 65 (45.8%)	127 (61.7%) 79 (38.3%)	0.167
Mode of admission	Self Referral	27 (19.0%) 115 (81.0%)	25 (12.1%) 181 (87.9%)	0.077

^a^ Single, divorced, and widowed PEWSF: pre-eclampsia with severe features *p*-values display a crosstabs chi-square test.

### Medical and reproductive history

In their most recent pregnancies, 114 (80.3%) of the women with favorable perinatal outcomes and 138 (67.0%) of the women with unfavorable outcomes received four to six antenatal care contacts (*p* = 0.000). The majority of women who had favorable outcomes, 138 (97.2%), and unfavorable outcomes, 188 (91.3%), gave birth to single kids (*p* = 0.019). Furthermore, a family history of hypertension was present in 18 (12.7%) of mothers with favorable outcomes and 52 (25.2%) with unfavorable outcomes (*p* = 0.004) (Table 2).

**Table 2:** Medical and reproductive history of mothers admitted with PEWSF at two selected public hospitals in Addis Ababa, Ethiopia, 2023.

Variables	Category	Perinatal outcomes		
		Favorable (142)	Unfavorable (206)	*p*-value
Gravidity	Primigravida Multigravida Grand multipara	69 (48.6%) 70 (49.3%) 3 (2.1%)	110 (53.4%) 92 (44.7%) 4 (1.9%)	0.678
Parity	Nulliparous 1–3 ≥4	3 (2.1%) 133 (93.7%) 6 (4.2%)	9 (4.4%) 189 (91.7%) 8 (3.9%)	0.522
History of abortion	Null 1 ≥ 2	115 (81.0%) 22 (15.5%) 5 (3.5%)	164 (79.6%) 39 (18.9%) 3 (1.5%)	0.342
Antenatal care (ANC) contact	Yes No	140 (98.6%) 2 (1.4%)	202 (98.1%) 4 (1.9%)	0.528
Number of ANC contact	1–3 4–6 7–8	15 (10.5%) 114 (80.3%) 13 (9.2%)	59 (28.6%) 138 (67.0%) 9 (4.4%)	0.000
Number of fetuses	Singleton Twin	138 (97.2%) 4 (2.8%)	188 (91.3%) 18 (8.7%)	0.019
History of PIH	Yes No	16 (11.3%) 126 (88.7%)	18 (8.7%) 188 (91.3%)	0.435
Family history of hypertension	Yes No	18 (12.7%) 124 (87.3%)	52 (25.2%) 154 (74.8%)	0.004
Past medical history	Yes No	11 (7.7%) 131 (92.3%)	16 (7.8%) 190 (92.2%)	0.994
Chronic hypertension	Yes No	3 (2.1%) 139 (97.9%)	9 (4.4%) 197 (95.6%)	0.257
Anemia	Yes No	5 (3.5%) 137 (96.5%)	3 (1.5%) 203 (98.5%)	0.184
Diabetes mellitus	Yes No	2 (1.4%) 140 (98.6%)	3 (1.5%) 203 (98.5%)	0.670
Renal disease	Yes No	1 (0.7%) 141 (99.3%)	1 (0.5%) 205 (99.5%)	0.650

PIH, pregnancy-induced hypertension, *p*-values display a crosstabs chi-square test.

### Clinical features and investigations on admission

About 63 (44.4%) of mothers with favorable and 117 (56.8%) unfavorable outcomes were admitted with a chief complaint of headache (*p* = 0.023). Most mothers with favorable, 112 (78.9%), and unfavorable outcomes, 185 (89.8%), had a severe blood pressure reading at admission (*p* = 0.005). Additionally, labor was induced in 86 (60.6%) of mothers with favorable and 158 (76.7%) unfavorable outcomes (*p* = 0.001). Further, on an investigation, 34 (16.5%), 22 (10.7%), 23 (11.2%), and 152 (73.8%) of mothers with unfavorable outcomes had deranged liver function test (*p* = 0.000), urea (*p* = 0.004), creatinine (*p* = 0.021), and urine protein on admission (*p* = 0.004), respectively (Table 3).

**Table 3:** Clinical features of mothers admitted with PEWSF at two selected public hospitals in Addis Ababa, Ethiopia, 2023.

Variables	Category	Perinatal outcomes		
		Favorable (142)	Unfavorable (206)	*p*-value
Headache	Yes No	63 (44.4%) 79 (55.6%)	117 (56.8%) 89 (43.2%)	0.023
Dizziness	Yes No	6 (4.2%) 136 (95.8%)	39 (18.9%) 167 (81.1%)	0.000
Epigastric pain	Yes No	44 (31.0%) 98 (69.0%)	75 (36.4%) 131 (63.6%)	0.295
Visual disturbance	Yes No	13 (9.2%) 129 (90.8%)	46 (22.3%) 160 (77.7%)	0.001
Nausea and/or vomiting	Yes No	4 (2.8%) 138 (97.2%)	11 (5.3%) 195 (94.7%)	0.194
Convulsion	Yes No	4 (2.8%) 138 (97.2%)	29 (14.1%) 177 (85.9%)	0.000
Edema	Yes No	32 (22.5%) 110 (77.5%)	55 (26.7%) 151 (73.3%)	0.378
Grade of edema (*n* = 87)	Grade 1 Grade 2 Grade 3	19 (59.4%) 13 (40.6%) 0 (0.0%)	27 (49.0%) 25 (45.5%) 3 (5.5%)	0.323
Blood pressure at admission	Severe range Mild range	112 (78.9%) 30 (21.1%)	185 (89.8%) 21 (10.2%)	0.005
Hematocrit	<33% ≥ 33%	15 (10.6%) 127 (89.4%)	24 (11.7%) 182 (88.3%)	0.752
Liver function test	Normal Deranged	138 (97.2%) 4 (2.8%)	172 (83.5%) 34 (16.5%)	0.000
Urea	Normal Deranged	138 (97.2%) 4 (2.8%)	184 (89.3%) 22 (10.7%)	0.004
Creatinine	Normal Deranged	136 (95.8%) 6 (4.2%)	183 (88.8%) 23 (11.2%)	0.021
Urine protein (Dipstick)	Negative 1+ 2+ 3+	51 (35.9%) 28 (19.7%) 48 (33.8%) 15 (10.6%)	54 (26.2%) 22 (10.7%) 92 (44.7%) 38 (18.4%)	0.004
Onset of labor	Spontaneous Induction	56 (39.4%) 86 (60.6%)	48 (23.3%) 158 (76.7%)	0.001
Mode of delivery	SVD Instrumental Cesarean section	71 (50.0%) 6 (4.2%) 65 (45.8%)	115 (55.8%) 8 (3.9%) 83 (40.3%)	0.562
Sex of the neonate	Male Female	79 (55.6%) 63 (44.4%)	107 (51.9%) 99 (48.1%)	0.497
Duration of hospital stay	≤3 days ≥4 days	81 (57.0%) 61 (43.0%)	54 (26.2% 152 (73.8%)	0.000

SVD, spontaneous vaginal delivery *p*-values display a crosstabs chi-square test.

### Perinatal outcomes

**Figure 1:**
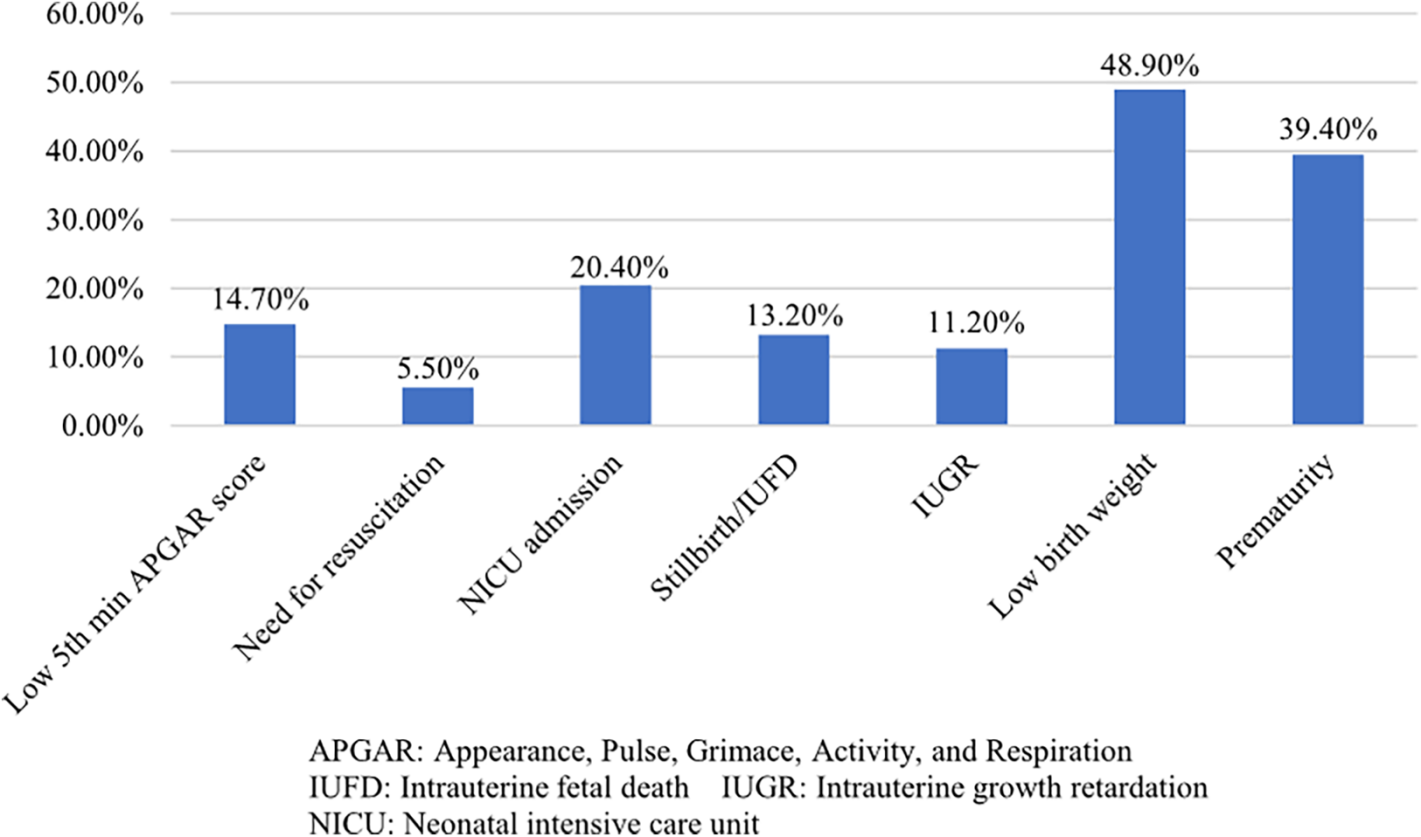
Adverse perinatal outcomes of mothers admitted with PEWSF at two selected public hospitals in Addis Ababa, Ethiopia, 2023.

The overall prevalence of unfavorable perinatal outcomes was 59.2% (*N* = 206) (95% CI: 54.0–63.8). Low birth weight 170/348 (48.9%), preterm 137/348 (39.4%), NICU hospitalization 71/348 (20.4%), and low fifth-min APGAR score 51/348 (14.7%) were frequently occurred unfavorable perinatal outcomes (Figure 1).

### Factors of unfavorable perinatal outcome

Bivariable logistic regression analysis was computed, and variables with a *p*-value of ≤0.25 were selected for the multivariable logistic regression analysis model. Accordingly, residence, level of education, marital status, occupation, mode of admission, number of ANC contacts, number of fetuses, and family history of hypertension were selected. In the final model, level of education, occupation, mode of admission, number of ANC contacts, and family history of hypertension have shown a statistically significant association with unfavorable perinatal outcomes.

Mothers who didn't attend formal education were five times more likely to develop unfavorable perinatal outcomes compared to those who attended higher education [OR = 5.14, 95% CI: (1.93–13.63)]. The odds of unfavorable perinatal outcomes were lower among unemployed mothers than the employed [OR = 0.42, 95% CI: (0.24–0.73). Unfavorable perinatal outcomes were two times more common among referred cases than self-admitted [OR = 2.03, 95% CI: (1.08–4.06)]. Mothers who have only 1–3 Antenatal care (ANC) contact were three times more likely to have unfavorable perinatal outcomes compared to those who have 7–8 contact [OR = 3.63, 95% CI: (1.22–10.71)]. Further, a family history of hypertension significantly increased the risk of unfavorable perinatal outcomes by approximately twofold [OR = 1.99, 95% CI: (1.03–3.85)] (Table 4).

**Table 4:** Factors associated with unfavorable perinatal outcome among mothers admitted with PEWSF at two selected public hospitals in Addis Ababa, Ethiopia, 2023.

Variables	Perinatal outcome		COR (95% CI)	AOR (95% CI)
	Favorable	Unfavorable		
Residence				
Urban	117 (82.4%)	155 (75.2%)	1	1
Rural	25 (17.6%)	51 (24.8%)	1.54 (0.90–2.63)	1.13 (0.61–2.09)
Level of education				
No formal education	10 (7.0%)	35 (17.0%)	3.50 (1.51–8.12)	5.14 (1.93–13.6)*
Primary	32 (22.5%)	65 (31.5%)	2.03 (1.09–3.80)	3.18 (1.47–6.89)*
Secondary	64 (45.1%)	70 (34.0%)	1.09 (0.62–1.94)	1.48 (0.77–2.82)
Higher education	36 (25.4%)	36 (17.5%)	1	1
Marital status				
Married	137 (96.5%)	187 (90.8%)	1	1
Others	5 (3.5%)	19 (9.2%)	2.78 (1.01–7.64)	1.44 (0.47–4.44)
Occupation				
Employed	77 (54.2%)	127 (61.7%)	1	1
Unemployed	65 (45.8%)	79 (38.3%)	0.74 (0.48–1.14)	0.42 (0.24–0.73)*
Mode of admission				
Self	27 (19.0%)	25 (12.1%)	1	1
Referral	115 (81.0%)	181 (87.9%)	1.70 (0.94–3.07)	2.03 (1.08–4.06)*
Number of ANC contact				
1–3	15 (10.5%)	59 (28.6%)	5.68 (2.05–15.78)	3.63 (1.22–10.7)*
4–6	114 (80.3%)	138 (67.0%)	1.75 (0.72–4.24)	1.08 (0.42–2.76)
7–8	13 (9.2%)	9 (4.4%)	1	1
Number of fetuses				
Singleton	138 (97.2%)	188 (91.3%)	1	1
Twin/multiple	4 (2.8%)	18 (8.7%)	3.30 (1.09–9.98)	3.01 (0.92–9.83)
Family history of hypertension				
Yes	18 (12.7%)	52 (25.2%)	2.33 (1.29–4.18)	1.99 (1.03–3.85)*
No	124 (87.3%)	154 (74.8%)	1	1

* Statistically significant at *p*-value < 0.05.

## Discussion

The overall prevalence of unfavorable perinatal outcomes was 59.2% (95% CI: 54.0–63.8). Level of education, occupation, mode of admission, number of ANC contacts, and family history of hypertension have shown a statistically significant association with unfavorable perinatal outcomes. Low birth weight (48.9%), preterm (39.4%), NICU hospitalization (20.4%), and low fifth-minute APGAR score (14.7%) were frequently occurred unfavorable birth outcomes.

Unfavorable perinatal outcomes occurred in 59.2% of mothers with preeclampsia with severe features. This is comparable with the study findings from Nigeria, where 63.5% of preeclamptic mothers have unfavorable perinatal outcomes (11). However, it was higher than 46.6% in Ethiopian referral hospitals (13). Variations in the research population, time, setup, sample size, and the caliber and standard of care offered by modern, well-equipped maternity hospitals, together with competent prenatal and obstetric care, could account for this disparity. On the other hand, this was lower than 66.4% in Addis Ababa, Ethiopia (14). Variations in the severity of the disease, variations in clinical features (severity signs and symptoms) at admission, study period, and gestational age at diagnosis could be the cause of variations in the incidence proportion of adverse outcomes between the studies. These results suggest that women with PEWSF in particular require greater improvements in the utilization and access to maternity services. This should be used in conjunction with adequate service delivery and early case detection and management to minimize unfavorable prenatal outcomes.

Mothers who didn't attend formal education were five times more likely to develop unfavorable perinatal outcomes compared to those who attended higher education. Socioeconomic conditions have also been recognized by the national policy as a critical area for reform (30). This is consistent with a study conducted in Amhara region referral hospitals (13), which found that babies delivered to mothers with low educational attainment were more likely to experience adverse perinatal outcomes. The possible reason could be attributed to the fact that mothers with higher educational status are more likely to enhance their health care-seeking behaviors, including having adequate antenatal care, which can lead to improved perinatal outcomes. Additionally, educated mothers may also be exposed to more health education messages and campaigns, enabling them to identify potential problems and danger indicators, and take the necessary precautions during pregnancy. To overcome this challenge, Ethiopia launched a health extension program in 2003 to promote preventive health actions including health education and awareness creation straight to the homes at the local level (30, 31). But more effort needs to be made at the community level.

Compared to working mothers, unemployed mothers were less likely to have negative outcomes. In the Netherlands, the mean birth weight of children dropped by 45 g when working mothers put in more hours (≥40 h/week) (32). Similarly, employed women in South Korea (33) had greater rates of stillbirths and early miscarriages. One explanation could be that women without jobs are more likely to have the time to take care of themselves and tune in to the latest information about pregnancy-induced hypertension on the radio, television, or other media. This could perhaps mitigate the likelihood of adverse consequences for them. In general, this result suggests that setting adequate time for self-care and adhering to health recommendations is important to reduce adverse perinatal outcomes.

According to this study, a woman's admittance status significantly influenced her perinatal outcome. Referred cases were twice as likely to have unfavorable perinatal outcomes as self-admitted ones. Similarly, a prospective study conducted in Zimbabwe examined the results of pregnancy-related referrals from rural health facilities. It found that there were nine maternal deaths, a 4.4% case fatality rate, and 151 perinatal mortality rates per 1,000 live births (34). Additionally, it was found that referral systems greatly increase the risk of maternal death in Ethiopia (35, 36). Most of the referred cases might have potentially complicated cases involving the central nervous system, coma or loss of consciousness lasting 12 h or more; metabolic coma (consciousness loss and urine containing ketoacids and glucose); stroke, status epilepticus, involuntary convulsions, or complete paralysis. In most cases, these abnormalities lead to unfavorable events. It could also be due to delayed referrals, inadequate early diagnosis of potentially life-threatening complications, lack of transportation, or being too far away from referral facilities (second delay). Overall, the finding implied that coordinated effort is needed to enhance the referral system including 24-h ambulance services and availing essential drugs and supplies to improve both maternal and perinatal outcomes.

Mothers who have only 1–3 Antenatal care (ANC) contacts were three times more likely to experience unfavorable perinatal events compared to those who have 7–8 contacts. Ethiopia is tackling significant health sector issues, such as low health service utilization and a lack of human resources for health, by implementing the novel Health Extension Programme (HEP), accelerating midwifery training, Integrated Emergency Surgery and Obstetrics task shifting, and expanding family planning (30). To provide a basic package of preventive and limited curative health care, including maternity and child health services, in urban, rural, and pastoral areas, the HEP trains health extension workers (23). To improve access to necessary services, the HEP has trained and deployed about 38,000 health workers, and about 16,000 health posts and 3,000 health centers have been built (30, 37). In Southeast Ethiopia (22), a similar finding was observed: women who skipped ANC follow-up were more likely to have adverse outcomes. In northern Ethiopia, lack of ANC significantly affected perinatal outcomes (38). Furthermore, a different study conducted in Ethiopia discovered that receiving optimal prenatal care could save perinatal deaths from cardiovascular and respiratory diseases and add 1 week to intrauterine life (39). Prenatal care follow-up promotes an opportunity for early screening and treatment of hypertensive disorders of pregnancy (HDP) and access to information related to the severity features of HDP. Additionally, mothers who receive prenatal care are more likely to give birth with the assistance of a trained birth attendant, which means that their babies will have access to basic medical treatment and neonatal resuscitation. Therefore, to lower the rate of unfavorable delivery outcomes and meet sustainable development goals, more work needs to be done to improve the quality of prenatal care and mobilize pregnant women to follow the new WHO recommendations for focused ANC treatment.

Further, a family history of hypertension significantly increased the risk of unfavorable perinatal outcomes by approximately twofold. This is comparable with the study done in Zanzibar (7). It revealed that a family history of high blood pressure was a risk factor for severe preeclampsia and adverse pregnancy outcomes. Similarly in Ethiopia, the likelihood of pregnancy-induced hypertension was predicted by a family history of the condition (40, 41). These results suggest that this disease may be caused by both maternal and fetal genes. Therefore, it should be closely monitored during pregnancy as well as in the postpartum period for women with a family history of HDP.

Despite the meticulous execution of our investigation, there are certain limitations. It has the same inability to establish a causal association as cross-sectional studies. Because this study was conducted in referral hospitals, we are unsure of whether these women delayed going to the primary health facilities or if there were delays in referring them. The predictive power of the model might be low given the small Nagelkerke R square value. Additionally, because the study was conducted in a hospital, the perinatal outcome for women who were delivered at home was not evaluated. Furthermore, adverse perinatal outcomes that occur after 24 h following birth are not included in this study.

## Conclusion

In this study, the prevalence of unfavorable perinatal outcomes was high compared to other studies in Ethiopia. Level of education, occupation, mode of admission, ANC contact, and family history of hypertension were significant predictors of unfavorable perinatal outcomes. Socio-economic development, improving referral systems, and adequate antenatal care contact are needed to improve unfavorable outcomes. Additionally, antenatal screening and specialized care for high-risk mothers, i.e., those with a family history of hypertension is recommended. Further long-term studies investigating the birth outcomes of women with severe pre-eclampsia symptoms are recommended.

## Data Availability

The raw data supporting the conclusions of this article will be made available by the authors, without undue reservation.
